# Ischemic heart disease-related mortality in Brazil, 2006 to 2020. A study of multiple causes of death

**DOI:** 10.1186/s12889-024-18162-0

**Published:** 2024-03-19

**Authors:** Luiz A. V. M. Bastos, Paolo B. Villela, Jose L. P. Bichara, Emilia M. do Nascimento, Eduardo L. V. M. Bastos, Basilio de B Pereira, Glaucia M. M. Oliveira

**Affiliations:** 1 Federal University of Rio de Janeiro, Rio de Janeiro, Brazil; 2 Rio de Janeiro State University, Rio de Janeiro, Brazil

**Keywords:** Cardiovascular diseases, Ischemic heart disease, Multiple causes of death, Death certificates

## Abstract

**Context:**

Both the aging of the population and the increase in noncommunicable diseases may influence the progression and outcomes culminating in death, changing the evolution of ischemic heart diseases (IHDs) and their associated causes. Using the multiple causes of death method could help understand the magnitude of these relationships and enable better targeting of investments in health.

**Objectives:**

To evaluate the mortality from IHD in Brazil between 2006 and 2020 using the method of multiple causes and identify differences in the distribution pattern of IHD mortality by sex and geographic region.

**Methods:**

Based on information extracted from death certificates (DCs) obtained from the database of the Department of Informatics of the Unified Health System (DATASUS), we used the multiple causes method to analyze the causes of death associated with IHD when IHD was defined as the underlying cause of death (UC) and the causes of death listed as the UC when IHD was recorded in any other lines of the DC, from 2006 to 2020, in Brazil. Subsequently, the proportion of these causes of death and differences between sexes and geographic regions were evaluated, with statistical relevance analyzed using the chi-square test, and the dependence between factors illustrated using stacked bar charts and small-world network graphs.

**Results:**

When IHD was listed as the UC of death, the most frequent associated causes of death were, in descending order of frequency, acute myocardial infarction (AMI), arterial hypertension (AH), chronic ischemic heart disease (CHID), heart failure (HF), and diabetes mellitus (DM). When IHD was mentioned in any line of the DC, the most frequent UCs of death were AMI followed by DM, CIHD, chronic obstructive pulmonary disease (COPD), stroke, dyslipidemia, and, in the year 2020, COVID-19. The most frequent cause of death in women were DM as the UC and associated cause of death, AH as the UC, and CIHD and Alzheimer's disease as associated causes of death, while the most frequent causes of death in men were substance dependence as the UC and associated cause of death, and cancer as an associated cause of death. The most frequent causes of death were DM and stroke in the North and Northeast, dyslipidemia and obesity in the Midwest, Alzheimer's disease in the South and Southeast, and atherosclerotic heart disease (AHD) and COPD in the South.

**Conclusions:**

Several diseases – including AMI, AH, CIHD, HF, and DM – were the most frequent associated causes of death when IHD was recorded as the UC. In contrast, AMI, DM, CIHD, COPD, and stroke were the most frequent UCs when IHD was listed as an associated cause of death. The degree of these associations varied between sexes and geographic regions.

**Supplementary Information:**

The online version contains supplementary material available at 10.1186/s12889-024-18162-0.

## Introduction

The evaluation of mortality based on the underlying cause (UC) of death enables the analysis of the cause that triggered the events that culminated in the death [1]. The evaluation of mortality using the method that considers multiple causes (MCs) of death expands this concept. This method investigates the sequence of events that occurred in association with the UC in addition to the associated causes by including in the analysis all causes mentioned in the death certificate (DC), that is, those present in lines B, C, and D of Part I and Part II [2, 3]. These data may be obtained from DCs but can also be collected from a review of medical records, diagnostic tests, and autopsy reports [3, 4]. The importance of the analysis by MCs has been known for more than 50 years, but this method is still scarcely studied, especially with regard to cardiovascular diseases (CVDs) [4].

From a global perspective, a classic Dutch study from the 1990s confirmed that analyses based on UCs tend to overestimate deaths due to certain etiologies and underestimate the importance of chronic associated diseases, *e.g.*, the weight of arterial hypertension (AH) as the most important associated cause of death in CVDs [3, 5]. A 2015 study from New York observed a higher prevalence of diabetes mellitus (DM) and inflammatory diseases among patients with premature death from ischemic heart disease (IHD); specifically, DM and inflammatory disease were five times more frequent in patients with premature death from IHD compared with individuals of the same age in the general population [6]. More recently, a 2020 Australian study using data from the United States and Australia and analysis with the MC method found that overweight and obesity were associated with a reversal in the downward trend in CVD mortality rates in these countries [7].

A 2007 Brazilian study based on data from 2003 also showed that analyses by UCs underestimate the mortality from chronic diseases, especially that from heart failure (HF) and AH [8]. In 2018, another Brazilian study, that gave rise to our project, based on 2004–2013 data showed that deaths due to AH increased by approximately 400% when the analysis was performed using the MC compared with the UC method [9]. A similar trend was observed in a 2021 study of this same project, in which deaths due to HF increased by almost 300% when the analysis used the MC instead of the UC method [10].

Few studies have analyzed the mortality from IHDs using the MC method over time to evaluate the contribution of other causes to deaths in which IHD was listed as the UC [6, 11–13]. With the aging of the population and presenting multiple comorbidities, it is essential to know the causes associated with ischemic heart disease so that we can prevent and treat them completely. Thus, the present study aimed to evaluate the mortality from IHD in Brazil between 2006 and 2020 using the MC method to identify differences in the occurrence of IHD deaths by sex and geographic region.

## Methods

This ecological study used data from DCs recorded between 2006 and 2020 in Brazil. The choice for this period was based on information from previous studies that showed improvement in the completion of DC since the early 2000s [14]; this was accomplished after the addition (in 1999) of another line in Part I to include more antecedent causes of death in the DC [3]. Further improvements in the completion of DCs came after a 2004 advertisement campaign by the Brazilian Ministry of Health to reduce the listing of deaths from ill-defined causes [15, 16].

The DC data were obtained from the Mortality Information System (*Sistema de Informações sobre Mortalidade*, SIM) available on an electronic site maintained by the Department of Informatics of the Unified Health System (*Departamento de Informática do Sistema Único de Saúde*, DATASUS) of the Brazilian Ministry of Health [17]. Data from 2006 to 2020 were obtained from all Brazilian federative units. The data were downloaded in their original.DBC format and converted to.DBF using the software Tabwin (DATASUS, Brazil). The data were then exported, converted to.CSV, and saved as.XLS in an Excel 2016 spreadsheet (Microsoft Corporation, Seattle, WA, USA). The.XLS format was then used throughout the study.

Next, a filter was applied for each year, and a formula was used to select the Excel rows in which the UCs had International Classification of Disease 10 (ICD-10) codes starting with I2, as detailed in Supplementary Material 1. Manual filtering of all Excel rows was then carried out consecutively to select only the rows in which the UCs had ICD-10 codes I20.0 to I25.9 (IHD group) and exclude the rest of the rows. After this selection, the 13 most frequent causes of death listed in any line when IHD was selected as the UC were identified in Brazil and subsequently stratified by sex and geographic region (North, Northeast, Midwest, Southeast, and South).

Subsequently, the degree of dependence between the causes of death, sexes, and geographic regions was evaluated using small-world networks (igraph package [Csardi & Nepusz, 2006] of R [R Core Team, 2021], in which the path thickness in the graphs is proportional to the number of links between the nodes [18].

For the analysis of the most listed causes of death, ICD-10 codes with similar clinical significance were grouped together. For example, Acute Ischemic Heart Disease (I24), Angina Pectoris (I20), and Acute Myocardial Infarction (I21) were grouped as "acute myocardial infarction" (AMI). Chronic Ischemic Heart Disease (I25) and Generalized Atherosclerosis (I70) were grouped as "atherosclerotic heart disease" (AHD). Heart Failure (I50), Cardiomyopathies (I42), and Pulmonary Edema (J81) were grouped as "heart failure" (HF). Infectious processes, represented by Pneumonia (J189 and J159) and Sepsis (A419), were grouped as "infection." Diabetes Mellitus (DM) was mentioned with several different ICD-10 codes (ICD-10 E10.0 to E14.9), thus requiring unification. After selection, we merged clinically similar codes. Finally, codes with small clinical significance that did not help define the cause of death, *e.g.*, Respiratory Arrest (R09), Respiratory Failure (J96), Cardiac Arrest (I46), Cardiac Arrhythmia (I49 and I48), Other General Symptoms and Signs (R68), Senility (R54), Unspecified Heart Disease (I51), Sudden Death (I46 and R96), and Ill-Defined Causes (R99), were grouped as "garbage codes" (GCs), *i.e.*, ill-defined causes that do not define a UC.

In a second moment, a new data filter was applied for each year, and the Excel rows in which the ICD-10 started with I2 in any line of the DC were selected using the formula described in Supplementary Material 2. This was followed by a manual selection of cases in which IHD (*i.e.*, ICD-10 I20.0 to I25.9) was listed in any of the lines (B, C, D, or Part II). Next, the 16 most frequent causes of death listed in the "UC" column when IHD was selected in lines B, C, D, or Part II were identified and further stratified by sex and region. Small-world network graphs were also used to evaluate the degree of dependence between these causes of death, sexes, and geographic regions [18, 19].

Table S1 shows the number of mentions per DC, by year, and geographic region. There was no relevant increase in the number of mentions per certificate in almost all Geographic Regions. It is also noted that the Southern Region has the lowest averages over time, while the central-western region has the highest averages for the number of mentions per DC.

Finally, in analyses by sex and geographic region, the chi-square test was used to assess the frequency in which the observed event deviated significantly or not (p value) from the frequency in which it was expected.

## Results

Table 1 presents the distribution by sex of the 13 causes of death most frequently associated causes listed in any line from DC when IHD (ICD-10 I20.0 to I25.9) was listed as the UC, and the 16 most frequent UCs of death listed when IHD was recorded in one of the lines of the DC. The associations were statistically significant, reflected by p values < 0.05. The most frequent associated causes in both sexes were AMI, HF, and AH. Also noteworthy was the frequency of GCs, which accounted for approximately 13% of the associated causes when IHD was coded as the UC.

**Table 1:** Associated Causes with Ischemic Heart Diseases and Underlying Causes related with Ischemic Heart Diseases in Brazil from 2006 to 2020 by sex. Distribution by sex of the 13 most frequent causes of death listed in death certificates when ischemic heart disease was recorded as the underlying cause of death ("Associated Causes") and the 16 most frequent causes of death listed as the underlying cause of death when ischemic heart disease was recorded in any line of the death certificates ("Underlying Causes") in Brazil from 2006 to 2020. The numbers correspond to the number of times each cause of death was mentioned in the death certificates and are shown in absolute values

		**Men**	**Women**	**p**
**Associated** **Causes**	**Total**	**653,285 (100%)**	**464,701 (100%)**	** < 0.0001**
	AH	98,079 (15.0%)	79,057 (17.0%)	< 0.0001
	AKD	2,066 (0.3%)	1,382 (0.3%)	< 0.0001
	AMI	246,893 (38.0%)	167,115 (35.9%)	< 0.0001
	CIHD	74,048 (11.3%)	48,677 (10.5%)	< 0.0001
	CKD	2,836 (0.4%)	1,618 (0.3%)	< 0.0001
	COPD	2,815 (0.4%)	1,653 (0.4%)	< 0.0001
	DEP	10,266 (1.6%)	2,572 (0.6%)	< 0.0001
	DM	12,015 (1.8%)	14,566 (3.1%)	< 0.0001
	GC	89,498 (13.7%)	6,2605 (13.5%)	< 0.0001
	HF	101,296 (15.5%)	74,318 (16.0%)	< 0.0001
	Infections	9,063 (1.4%)	7,186 (1.5%)	< 0.0001
	PE	1,495 (0.2%)	1,634 (0.4%)	0.013
	Stroke	2,915 (0.4%)	2,318 (0.5%)	< 0.0001
**Underlying** **Causes**	**Total**	**315,223 (100%)**	**224,548 (100%)**	** < 0.0001**
	AKD	490 (0.2%)	270 (0.1%)	< 0.0001
	Alzheimer's	401 (0.1%)	540 (0.2%)	< 0.0001
	AMI	240,339 (76.1%)	163,779 (73.2%)	< 0.0001
	CA	1,565 (0.5%)	526 (0.2%)	< 0.0001
	ChD	908 (0.3%)	534 (0.2%)	< 0.0001
	CIHD	28,495 (9.0%)	22,212 (9.9%)	< 0.0001
	CKD	851 (0.3%)	472 (0.2%)	< 0.0001
	COPD	4,125 (1.3%)	2,728 (1.2%)	< 0.0001
	COVID-19	551 (0.2%)	302 (0.1%)	< 0.0001
	DEP	3,017 (1.0%)	210 (0.1%)	< 0.0001
	DLP	2,260 (0.7%)	1,672 (0.7%)	< 0.0001
	DM	22,367 (7.1%)	23,416 (10.4%)	< 0.0001
	HF	3,677 (1.2%)	1,806 (0.8%)	< 0.0001
	I	1,279 (0.4%)	1,917 (0.9%)	< 0.0001
	Obesity	1,177 (0.4%)	1,452 (0.6%)	< 0.0001
	Stroke	3,721 (1.2%)	2,712 (1.2%)	< 0.0001

*Abbreviations:* *AH* arterial hypertension, *AKD* acute kidney disease, Alzheimer's Alzheimer's disease, *AMI* acute myocardial infarction, *CA* cancer, *ChD* Chagas' disease, *CIHD* chronic ischemic heart disease, *CKD* chronic kidney disease, COPD chronic obstructive pulmonary disease, *COVID-19* coronavirus disease 2019, *DEP* substance dependence, *DLP *dyslipidemia, *DM* diabetes mellitus, *GC* garbage code, *HF* heart failure, *I* infections, *PE* pulmonary embolism

Table 2 shows the distribution by geographic region of the 13 associated causes of death most listed in any line from the DC when IHD (ICD-10 I20.0 to I25.9) was listed as the UC of death ("Associated Causes") and the 16 most frequent UCs of death listed when IHD was recorded in one of the lines of the DC ("Underlying Causes"). The frequency of the distribution of all causes of death varied significantly across geographic regions (p < 0.0001 for all). Notably, AMI, HF, and AH presented the highest rates, and GCs were frequent in all regions. The most frequent associated causes per geographic region were AH and AMI in the North and Northeast; DM in the Northeast; GC in the North and Midwest; CIHD, DM, and HF in the Midwest and Southeast; and stroke, CIHD, COPD, and infections in the South. The most frequent UCs of death listed when IHD was recorded in one of the lines of the DC were AMI, DM, and stroke in the North and Northeast regions; COVID-19, AKD, CKD, and infections in the North; DEP in the North and Midwest; ChD and dyslipidemia in the Midwest; HF in the Midwest and South; Alzheimer's disease and CKD in the Southeast and South; infections in the Southeast; and stroke, CIHD, and COPD in the South.

**Table 2:** Associated Causes with Ischemic Heart Diseases and Underlying Causes related with Ischemic Heart Diseases in Brazil from 2006 to 2020 by geographic region. Distribution by geographic region of the 13 most frequent causes of death listed in death certificates when ischemic heart disease was recorded as the underlying cause of death ("Associated Causes") and the 16 most frequent causes of death listed as the underlying cause of death when ischemic heart disease was recorded in any line of the death certificates ("Underlying Causes") in Brazil from 2006 to 2020. The numbers correspond to the number of times each cause of death was mentioned in the death certificates and are shown in absolute values

		**North**	**Northeast**	**Midwest**	**Southeast**	**South**	**p**
**Associated** **Causes**	**Total**	**121,714 (100%)**	**448,760 (100%)**	**191,793 (100%)**	**214,795 (100%)**	**157,710 (100%)**	** < 0.0001**
	AH	20,732 (17.0%)	79,543 (17.7%)	28,483 (14.9%)	32,833 (15.3%)	21,165 (13.4%)	< 0.0001
	AKD	494 (0.4%)	1,016 (0.2%)	775 (0.4%)	598 (0.3%)	562 (0.4%)	< 0.0001
	AMI	47,603 (39.0%)	170,506 (38.0%)	64,810 (33.8%)	77,305 (36.0%)	56,018 (35.4%)	< 0.0001
	CIHD	7,876 (6.5%)	43,568 (9.7%)	21,248 (11.1%)	26,714 (12.4%)	22,647 (14.4%)	< 0.0001
	CKD	560 (0.5%)	1,224 (0.3%)	938 (0.5%)	1,104 (0.5%)	616 (0.4%)	< 0.0001
	COPD	416 (0.3%)	1,051 (0.2%)	1,012 (0.5%)	841 (0.4%)	1,251 (0.8%)	< 0.0001
	DEP	810 (0.7%)	5,442 (1.2%)	2,948 (1.5%)	2,109 (1.0%)	1,913 (1.2%)	< 0.0001
	DM	2,768 (2.3%)	11,110 (2.5%)	4,852 (2.5%)	5,656 (2.6%)	3,638 (2.3%)	< 0.0001
	GC	19,268 (15.8%)	57,798 (12.9%)	30,816 (16.1%)	27,907 (13.0%)	22,804 (14.5%)	< 0.0001
	HF	18,322 (15.1%)	69,488 (15.5%)	31,241 (16.3%)	34,446 (16.0%)	22,671 (14.4%)	< 0.0001
	Infections	2,034 (1.7%)	4,736 (1.1%)	3,547 (1.8%)	3,614 (1.7%)	3,031 (1.9%)	< 0.0001
	PE	223 (0.2%)	1037 (0.2%)	424 (0.2%)	853 (0.4%)	523 (0.3%)	< 0.0001
	Stroke	608 (0.5%)	2241 (0.5%)	699 (0.4%)	815 (0.4%)	871 (0.6%)	< 0.0001
**Underlying** **Causes**	**Total**	**58,635 (100%)**	**216,439 (100%)**	**84,742 (100%)**	**100,590 (100%)**	**79,376 (100%)**	** < 0.0001**
	AKD	156 (0.3%)	288 (0.1%)	109 (0.1%)	115 (0.1%)	97 (0.1%)	< 0.0001
	Alzheimer's	56 (0.1%)	299 (0.1%)	109 (0.1%)	259 (0.3%)	221 (0.3%)	< 0.0001
	AMI	45,872 (78.2%)	166,822 (77.2%)	62,712 (74.4%)	74,905 (74.4%)	55,674 (70.0%)	< 0.0001
	CA	179 (0.3%)	467 (0.2%)	292 (0.3%)	293 (0.3%)	357 (0.4%)	< 0.0001
	ChD	84 (0.1%)	558 (0.3%)	550 (0.6%)	203 (0.2%)	45 (0.1%)	< 0.0001
	CIHD	3,402 (5.8%)	15,913 (7.4%)	8,813 (10.4%)	10,788 (10.7%)	11,651 (14.7%)	< 0.0001
	CKD	165 (0.3%)	436 (0.2%)	203 (0.2%)	299 (0.3%)	212 (0.3%)	< 0.0001
	COPD	757 (1.3%)	1,731 (0.8%)	1,211 (1.4%)	1,178 (1.2%)	1,566 (2.0%)	< 0.0001
	COVID-19	234 (0.4%)	308 (0.1%)	175 (0.2%)	115 (0.1%)	21 (0.0%)	< 0.0001
	DEP	190 (0.3%)	1,122 (0.5%)	526 (0.6%)	282 (0.3%)	352 (0.4%)	< 0.0001
	DLP	240 (0.4%)	1,495 (0.7%)	865 (1.0%)	787 (0.8%)	554 (0.7%)	< 0.0001
	DM	5,249 (9.0%)	20,349 (9.4%)	6,301 (7.4%)	7,538 (7.5%)	6,354 (8.0%)	< 0.0001
	HF	482 (0.8%)	1.941 (0.9%)	1,030 (1.2%)	1,280 (1.3%)	696 (0.9%)	< 0.0001
	Infections	475 (0.8%)	748 (0.3%)	456 (0.5%)	945 (0.9%)	367 (0.5%)	< 0.0001
	Obesity	219 (0.4%)	871 (0.4%)	615 (0.7%)	524 (0.5%)	444 (0.6%)	< 0.0001
	Stroke	875 (1.5%)	3,091 (1.4%)	775 (0.9%)	1,079 (1.1%)	765 (1.0%)	< 0.0001

*Abbreviations:* *AH* arterial hypertension, *AKD* acute kidney disease, Alzheimer's, Alzheimer's disease, *AMI* acute myocardial infarction, *CA* cancer, *ChD* Chagas' disease, *CIHD* chronic ischemic heart disease, *CKD* chronic kidney disease, *COPD* chronic obstructive pulmonary disease, *COVID-19* coronavirus disease 2019, *DEP* substance dependence, *DLP* dyslipidemia, *DM* diabetes mellitus, *GC* garbage code, *HF* heart failure, *PE* pulmonary embolism

**Fig. 1:**
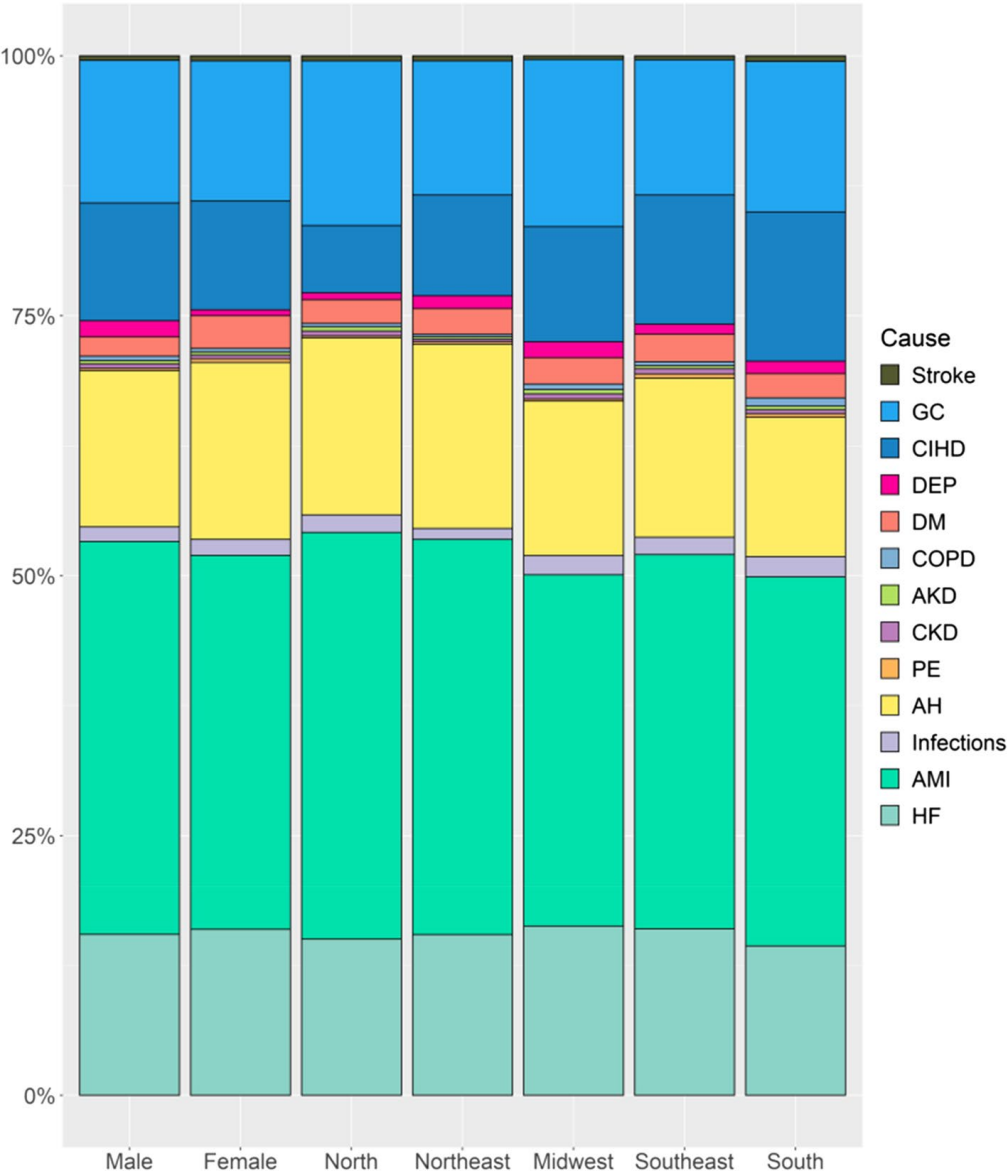
Associated Causes with Ischemic Heart Diseases by sex and geographic region in Brazil from 2006 to 2020. Stacked bar graph showing the associate causes of death related with ischemic heart disease in Brazil from 2006 to 2020, stratified by sex and geographic region. The colors are individualized for each of the 13 most frequent associated causes listed when ischemic heart disease was recorded as the underlying cause. Abbreviations: AH, arterial hypertension; AKD, acute kidney disease; AMI, acute myocardial infarction; CIHD, chronic ischemic heart disease; CKD, chronic kidney disease; COPD, chronic obstructive pulmonary disease; DEP, substance dependence; DM, diabetes mellitus; GC, garbage code; HF, heart failure; PE, pulmonary embolism

The stacked bar chart in Fig. 1 shows the proportional mortality of the 13 most frequent associated causes listed when IHD was recorded as the UC, stratified by sex and geographic region. The colors, individualized for each associated cause, indicate a similar distribution pattern between the associated causes and a higher proportional frequency of AMI, followed by HF. Between sexes, the associated causes most frequently listed were AH and DM in women and DEP in men. In the analysis by geographic regions, the associated causes more frequently listed were AH in the North and Northeast, DEP in the Midwest, CIHD in the South and Southeast, and COPD in the South.

**Fig. 2:**
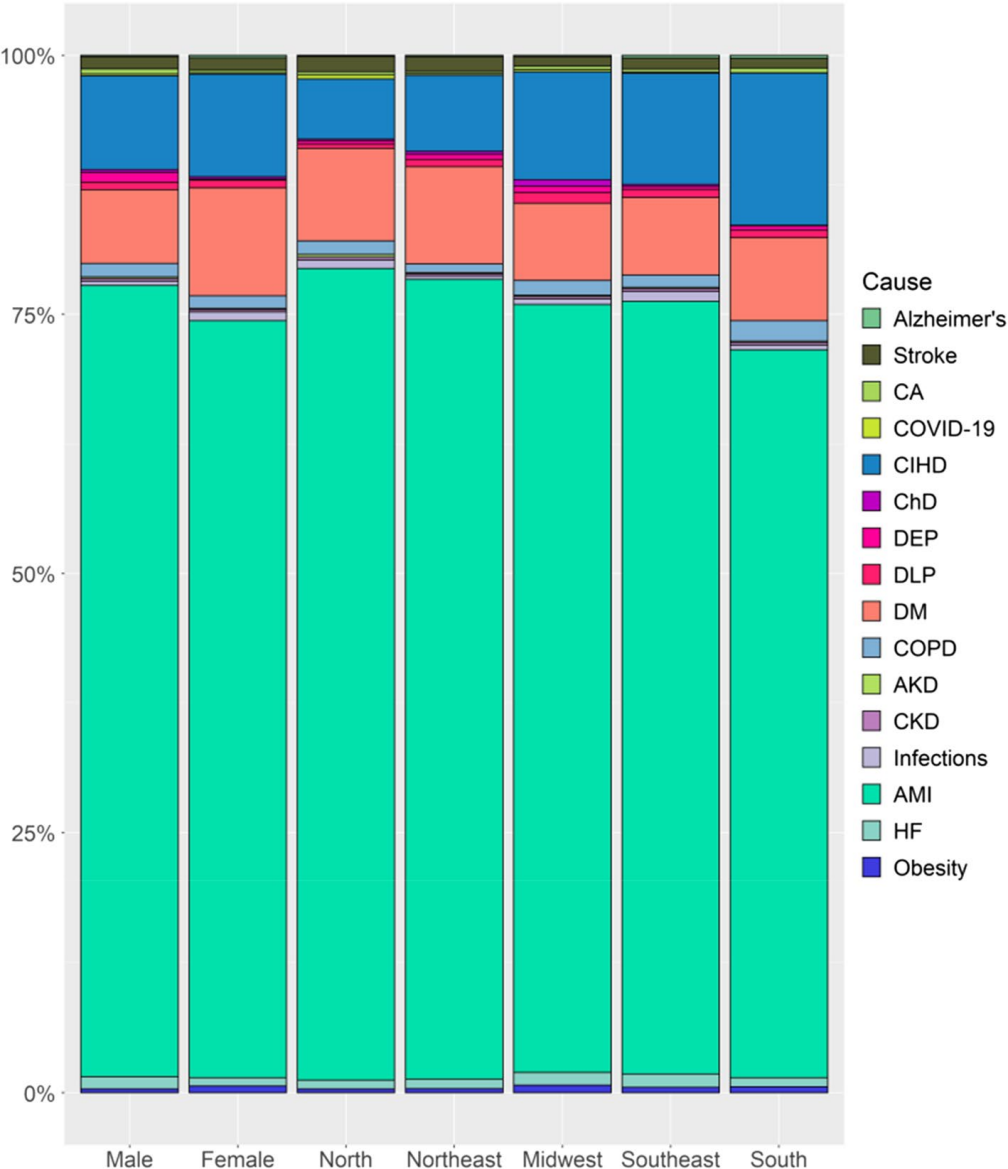
Underlying Causes of death associated with Ischemic Heart Diseases by sex and geographic region in Brazil from 2006 to 2020. Stacked bar chart showing the underlying causes of death associated with ischemic heart disease in Brazil from 2006 to 2020, stratified by sex and geographic region. The colors are individualized for each of the 16 most frequent underlying causes listed when ischemic heart disease was recorded in one of the lines of the death certificate. Abbreviations: AKD, acute kidney disease; Alzheimer's, Alzheimer's disease; AMI, acute myocardial infarction; CA, cancer; ChD, Chagas' disease; CIHD, chronic ischemic heart disease; CKD, chronic kidney disease; COPD, chronic obstructive pulmonary disease; COVID-19, coronavirus disease 2019; DEP, substance dependence; DLP, dyslipidemia; DM, diabetes mellitus; HF, heart failure

Figure 2 shows the 16 most frequent UCs reported when IHD was listed as associate causes of death, stratified by sex and geographic region. The proportional frequency was highest for AMI across all geographic regions and in both sexes, followed by DM and AHD. Between sexes, the most frequent UCs when IHD was listed in one of the lines of the DC were DM and stroke in women and DEP, HF, and cancer in men. Across geographic regions, the frequency was highest for DM and stroke in the North and Northeast; dyslipidemia and HF in the Midwest; obesity and CKD in the Southeast; and Alzheimer's disease, CIHD, and COPD in the South. During 2020, a short period of the analysis, COVID-19 emerged as one of the most cited UCs, with the highest proportion in the North region. Considering only the last year of the analysis (2020) and DCs in which IHD was listed in one of the lines of the DC, COVID-19 was listed as the UC of death in 2.4% of the DCs in the country and in 5.1% of those in the North, 2.3% in the Northeast, 2.8% in the Midwest, 2% in the Southeast, and 0.5% in the South.

**Fig 3 a and b:**
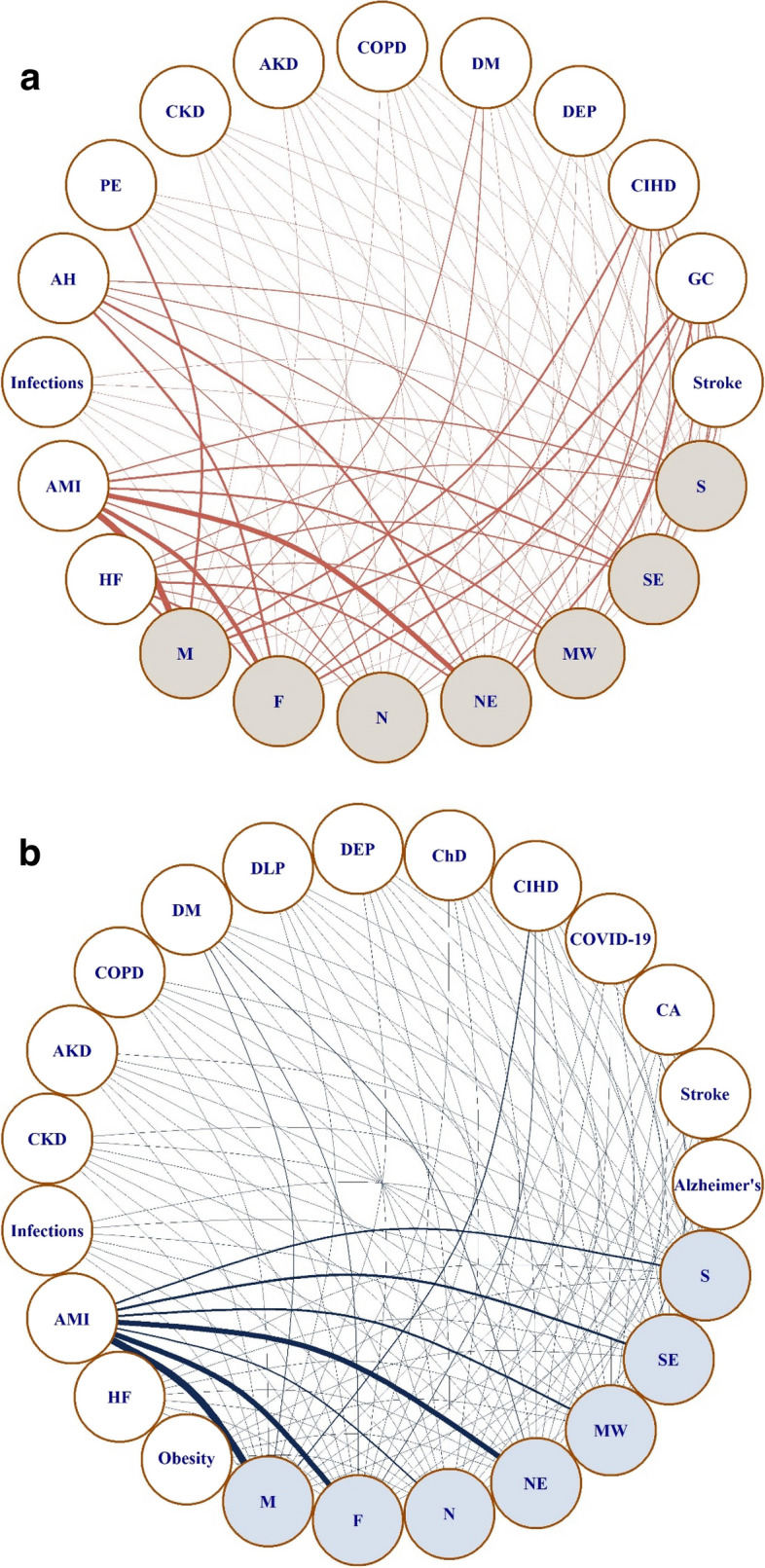
Small-world network models interconnecting Associated Causes with Ischemic Heart Diseases and Underlying Causes related with Ischemic Heart Diseases with sex and geographic regions. Graph representation of small-world network models of (3) the 13 most frequent associated causes of death listed when IHD was recorded as the underlying cause of death and (4) the 16 most frequent causes of death listed as the underlying cause of death when ischemic heart disease was recorded in any line of the death certificate. Both (3) and (4) are stratified by sex and geographic region. The thickness of the path is proportional to the number of links between nodes. Abbreviations: AH, arterial hypertension; AKD, acute kidney disease; Alzheimer's, Alzheimer's disease; AMI, acute myocardial infarction; CA, cancer; ChD, Chagas' disease; CIHD, chronic ischemic heart disease; CKD, chronic kidney disease; COPD, chronic obstructive pulmonary disease; COVID-19, coronavirus disease 2019; DEP, substance dependence; DLP, dyslipidemia; DM, diabetes mellitus; F, female sex; GC, garbage code; HF, heart failure; M, male sex; MW, Midwest region; N, North region; NE, Northeast region; PE, pulmonary embolism; S, South region; SE, Southeast region.

The small-world graphs in Fig. 3 a and b show the degree of relationship between IHD as the UC (Fig. 3a and b) and as MCs. The degree of relationship is illustrated by the thickness of the path connecting two nodes ("vertices"), which is proportional to the number of connections between the nodes. This depiction displays the most listed associated causes of death and their relationships between sexes and geographic regions. In Fig. 3, we observe the 13 most frequent associated causes of death listed when IHD was recorded as the UC and their associations with sex and geographic region. As shown, AMI was, proportionally, the most frequently listed associated cause of death in both men and women and in the Northeast region. Other associated causes of death with strong relationship with each sex were PE and CIHD in men and DM in women. Notably, AH was proportionally more associated with the male sex and the Northeast region. Fig 3a and b shows the same relationship with the 16 causes of death listed as the UC when IHD was recorded in any line of the DC. Likewise, the strongest association was with AMI in men and in the Northeast region. Notably, there was a high frequency of mentions of CIHD in the male sex and DM in the female sex.

## Discussion

Several diseases – including AMI, AH, CIHD, HF, and DM – were the most frequent associated causes of death when IHD was recorded as the UC. In contrast, AMI, DM, CIHD, COPD, and stroke were the most frequent UCs when IHD was listed as an associated cause of death. The degree of these associations varied between sexes and geographic regions.

The results of the present study showed that AMI is the cause of death listed most frequently when IHD is recorded as the UC or one of the MCs, as previously demonstrated [12]. Besides AMI, the other most frequent associated causes were AH, CIHD, HF, and DM, which had a similar distribution between sexes. Specifically, between sexes, the most frequent associated causes of death were DEP in men and DM and AH in women. The relevance of AH as an associated cause of CVD was pointed out by Villela et al*.* in a 2018 Brazilian study and by Santo et al. in a 2011 study on the association of cerebrovascular diseases and AH, which concluded that blood pressure control is associated with a reduction in deaths from IHD and cerebrovascular diseases. [9, 20, 21]

The importance of DM as an associated cause when IHD is listed as the UC was reported in a 2015 Italian study. Fedeli et al*.* observed that the frequency in which DM was listed increased by up to five times when the authors analyzed DM listed in any line of the DC compared with this cause of death listed only as the UC [5]; this finding is aligned with previous publications showing that DM is underreported when the analysis is limited to the UC [22, 23]. An American study by Quinone et al*.*confirmed the same finding with IHD, particularly in women and younger individuals [6].

In the analysis by geographic regions in the present study, AH was listed most frequently in the North region. These data corroborate a finding by Ishitani et al*.*, who demonstrated a significant increase in AH mentions when the analysis considering AH as an associated cause of death instead of the UC [3]. The high number of mentions of GCs reflects an inadequate completion of the DC that hinders the analysis of mortality in Brazil. Using data from the 2019 Global Burden of Disease, Johnson et al*.*mentioned in 2021 that GCs are frequent and harmful to DC analyses and inversely proportional to the social indicators of a community [24]. These codes were proportionally less frequent in the North and Northeast regions, suggesting an improvement in DC completion in these regions [15, 16, 25, 26].

The UCs most frequently associated with IHD when IHD was listed in at least one line of the DC were AMI, DM, CIHD, COPD, stroke, and dyslipidemia. The most frequently listed UCs in this context were DM, CIHD, and Alzheimer's disease among women and DEP and CA in men. Of note, DM is considered one of the main cardiovascular risk factors, and the relationship between DM and IHD has been known for many years, as has been the relevance of DM in the female sex [27–29]. The results of the present study indicate that IHD may be underestimated in analyses that consider DCs reporting DM only as the UC. The same bias has been observed when COPD, stroke, and dyslipidemia (which share similar risk factors and pathophysiology with IHD) have been each reported as the UC [7, 30, 31]. A similar finding was reported by Santo et al*.* in a 2022 Brazilian study with data from 2000 to 2019 [32]. In an analysis by MC, the authors observed that circulatory diseases were the most frequent causes of death associated with deaths from COPD as the UCs, both of which share smoking as an important risk factor, and confounding factors such as age. The association of CVDs with obesity was also reported by Adair et al*.*in a 2020 Australian study using the MC method; the authors found an increased mortality from CVD as associated cause, and DM, CKD, AH, and dyslipidemia as UCs, as found in the present work [7]. Additionally, a 2004 Brazilian study suggested an association of IHD with obesity, CVD, dyslipidemia, and AH as UCs [33].

In the present analysis by geographic regions, a greater importance of DM and stroke as UCs was observed in the North and Northeast regions, while the Midwest presented proportionally more mentions of dyslipidemia and obesity. These findings corroborate those of a study with data from France, Italy, and the United States, which observed an increased number of mentions of obesity when the MC method was used compared with the UC method [34]. The Southeast and South regions had a greater number of mentions of Alzheimer's disease, which is in line with a 2016 study that suggested an increase of up to 20% in listed mental illnesses (including dementias) when analyzed by MC [35]. These data may indicate better social indicators and longer life expectancy associated with dementia in these last two regions [13]. There was a predominance of CIHD and COPD in the South, which is the region with the highest proportional consumption of tobacco in the country [36]. The predominance of ChD in the Midwest region confirms data from other Brazilian studies, which have shown that this cause of death is underreported in DCs and has IHD as one of its associated causes along with arrhythmias, HF, CVD, and AH [37, 38].

During 2020, a short period in our analysis, COVID-19 was among the main UCs to which IHD was listed as an associated cause; this finding was more frequent in the North region. In 2020, COVID-19 was frequently listed as a UC when IHD was recorded as an associate cause. Notably, COVID-19 ranked fifth among the most relevant causes of death in this period, reinforcing the importance of this disease in worsening the prognosis of patients with IHD [39, 40]. A study by the Brazilian Ministry of Health that used the MC method to analyze the occurrence of CVD when COVID-19 was listed as the UC also observed an increased number of deaths from CVD in this period; as reported in the present study, this finding was also more frequent in the North region of Brazil [41]. These data corroborate the results of a 2022 Brazilian study that highlighted the importance of comorbidities in patients with COVID-19 as an associated cause of death, with the most cited comorbidities being DM, AH, CKD, obesity, and IHD [42].

The fact that mortality analyses rely on the quality of completion of the DC is a limitation of the present study. Since physicians complete the DC according to their knowledge and experience, the DCs could have presented biases and errors depending on the professionals' qualifications and training. Another relevant point is that the format of the DC, which is standardized worldwide, is designed with the main objective of identifying the UC of death and its course; this may discourage professionals from listing previous diseases and risk factors that may have influenced the deaths, thus compromising the analysis of mortality by the MC method [3, 4]. Another limitation of the present study is its observational design, which hinders the assessment of causality.

Future studies are needed for a better understanding of the associations found in the present study and to help guide investments in public health to reduce IHD mortality. There is also a need to further elucidate the differences between sexes and geographic regions to make investments more specific and personalized, yielding better results.

## Conclusion

The analysis of deaths from IHD using the MC method reinforced the relevance of this method and identified many causes of death associated with IHD and causes of death with which IHD is associated. Several causes of death, including AMI, AH, AHD, HF, and DM, were associated with IHD when IHD was listed as the UC. On the other hand, IHD was associated with AMI, DM, AHD, COPD, and stroke when these causes of death were listed as the UC. The degree of these associations varied between sexes and across geographic regions. Investments in resources to diagnose and treat these diseases can reduce the number of deaths from IHD, just as better care for IHD can lead to a reduction in deaths from DM, COPD, stroke, dyslipidemia, pneumonia, and obesity.

### Supplementary Information

**Supplementary Material 1.**

**Supplementary Material 2.**

**Supplementary Material 3.**

**Supplementary Material 4.**

**Supplementary Material 5.**

## Data Availability

All data generated or analysed during this study are included in this published article and its supplementary information files.

## Abbreviations

AH: Arterial hypertension
AHD: Atherosclerotic heart disease
AHD: Acute kidney disease
AMI: Acute myocardial infarction
CA: Cancer
CIHD: Chronic ischemic heart disease
ChD: Chagas' disease
CKD: Chronic kidney disease
COPD: Chronic obstructive pulmonary disease
COVID-19: Coronavirus disease 2019
CVD: Cardiovascular disease
DATASUS: *Departamento de Informática do Sistema Único de Saúde* (Department of Informatics of the Unified Health System)
DC: Death certificate
DEP: Substance dependence
DLP: Dyslipidemia
DM: Diabetes mellitus
F: Female sex
GC: Garbage code
HF: Heart failure
I: Infections
ICD-10: International Classification of Disease 10
IHD: Ischemic heart disease
M: Male sex
MC: Multiple cause
MW: Midwest region
N: North region
NE: Northeast region
PE: Pulmonary embolism
S: South region
SE: Southeast region
SIM: *Sistema de Informações sobre Mortalidade* (Mortality Information System)
UC: Underlying cause
