# Supplementary material for: Ischemic heart disease-related mortality in Brazil, 2006 to 2020. A study of multiple causes of death
Source: BMC Public Health. 2024 Mar 19;24:849. doi: 10.1186/s12889-024-18162-0 (PMC10949584; doi:10.1186/s12889-024-18162-0)
Supplement: Supplementary file 1 — Supplementary Material 1. [file 12889_2024_18162_MOESM1_ESM.pdf]

Supplementary Material 1.

The files of each federative unit were separated into folders by year, and the following code was then applied:

```
Sub Macro1()
'
 Dim xFile As String

    xFile = Dir("C:\Users\EBastos\Desktop\Trabalho Luiz\2020" & "\*.xls")

    Do While xFile <> ""
        Workbooks.Open "C:\Users\EBastos\Desktop\Trabalho Luiz\2020" & "\" & xFile
        xFile = Dir
        arquivo = ActiveWorkbook.Name

        Selection.End(xlDown).Select
        contador = ActiveCell.Row
        Selection.End(xlUp).Select

        Range("CJ1").Select
        ActiveCell.FormulaR1C1 = "Nome do Arquivo"

        For i = 2 To contador
```

```
Cells(i, 88).Value = ActiveWorkbook.Name
Next

Range(Selection, Selection.End(xlToLeft)).Select
Range(Selection, Selection.End(xlToRight)).Select
Range(Selection, Selection.End(xlDown)).Select
Selection.AutoFilter

Selection.AutoFilter Field:=46, Criteria1:="=i2*" _
, Operator:=xlFilterValues

Range("A1").Select
Range(Selection, Selection.End(xlToRight)).Select
Range(Selection, Selection.End(xlDown)).Select
Selection.Copy
ThisWorkbook.Activate
ActiveSheet.Select

Selection.PasteSpecial Paste:=xlPasteValues, Operation:=xlNone, SkipBlanks _
    :=False, Transpose:=False

Dim xCell As Range
For Each xCell In ActiveSheet.Columns(1).Cells
    If Len(xCell) = 0 Then
```

```
            xCell.Select
            Exit For
        End If
    Next
    Application.DisplayAlerts = False
    Workbooks(arquivo).Close
    Application.DisplayAlerts = True
Loop
End Sub
```

The first two bold highlights were replaced with the paths to the folders in which the files of the federative units corresponding to that specific year were saved. The third bold highlight corresponded to the first cell of the first column after the data. The fourth bold highlight corresponded to the total number of columns with data plus 1. The fifth term bold highlight corresponded to the number of the column where the underlying cause information that had to be filtered was located. The code opened each federative unit file for that year, filtered the underlying causes starting with "I2," copied all filtered data, and pasted them into a consolidated file. At the end of the entire process, there were 15 files remaining, one for each year, all of which displayed data for underlying causes beginning with "I2" for all federative units.

When the process was complete, a final data cleaning was performed. To accomplish that, the rows with underlying causes ranging from I20.0 to I25.9 were selected, and all the other rows were excluded. Following that, the asterisks of the associated causes (*i.e.*, those causes associated with the underlying cause) were excluded. Removing the asterisks was critical, as they would confuse the Excel formulas and generate duplicate calculations. Before excluding the asterisks, the initial asterisks (first letter) in the five columns of the associated causes were excluded. For that, the following formula was used: IFERROR(RIGHT(CELL;LEN(CELL)-1;””), where CELL was the cell in which the first asterisk had to be cleared. Then, the “replace” function was used to

replace the asterisk term with a space. It is important to note that a tilde ("~") must be inserted before the asterisk for this replacement to occur correctly. With this, the data will be correct and ready for the creation of formulas to start the analysis of recurrences of causes and associated causes.
