# Supplementary material for: Ischemic heart disease-related mortality in Brazil, 2006 to 2020. A study of multiple causes of death
Source: BMC Public Health. 2024 Mar 19;24:849. doi: 10.1186/s12889-024-18162-0 (PMC10949584; doi:10.1186/s12889-024-18162-0)
Supplement: Supplementary file 2 — Supplementary Material 2. [file 12889_2024_18162_MOESM2_ESM.pdf]

Supplementary Material 2.

A second code, indicated below, was applied to the files of each federative unit, which were separated into folders by year, in order to carry out a deeper analysis of the multiple causes.

```
Sub Macro1()
    Dim xFile As String
    xFile = Dir("C:\Users\EBastos\Desktop\Trabalho Luiz\2020" & "\*.xls")
    Do While xFile <> ""
        Workbooks.Open "C:\Users\EBastos\Desktop\Trabalho Luiz\2020" & "\" & xFile
        xFile = Dir
        arquivo = ActiveWorkbook.Name
        Selection.End(xlDown).Select
        contador = ActiveCell.Row
        Selection.End(xlUp).Select
        Range("CJ1").Select
        ActiveCell.FormulaR1C1 = "Nome do Arquivo"
        For i = 2 To contador
        Cells(i, 88).Value = ActiveWorkbook.Name
        Next
        Call Macro2
        Range("A1").Select
        Range(Selection, Selection.End(xlToRight)).Select
        Range(Selection, Selection.End(xlDown)).Select
        Selection.Copy
```

```
        ThisWorkbook.Activate
        ActiveSheet.Select
        Selection.PasteSpecial Paste:=xlPasteValues, Operation:=xlNone, SkipBlanks _
            :=False, Transpose:=False
        Dim xCell As Range
        For Each xCell In ActiveSheet.Columns(1).Cells
            If Len(xCell) = 0 Then
                xCell.Select
                Exit For
            End If
        Next
        Application.DisplayAlerts = False
        Workbooks(arquivo).Close
        Application.DisplayAlerts = True
    Loop
End Sub

Sub Macro2()
    Selection.End(xlToRight).Select
    Range("CL2").Select
    ActiveCell.FormulaR1C1 = _
        "=IFERROR(IF(LEFT(RIGHT(RC[-49],LEN(RC[-49])-1),2)=""I2"",1,0),"""")"
    Range("CL2").Select
```

Selection.Copy

Range("**CL2:CP2**").Select

ActiveSheet.Paste

Range("**CL2**").Select

Selection.End(xlToLeft).Select

Selection.End(xlDown).Select

ActiveCell.Offset(0, 2).Select

Range(Selection, Selection.End(xlUp)).Select

ActiveSheet.Paste

Columns("**CL:CL**").Select

Range("**CL2**").Activate

Application.CutCopyMode = False

Selection.Copy

Columns("**CL:CP**").Select

Range("**CL2**").Activate

ActiveSheet.Paste

Range("**CL1**").Select

Application.CutCopyMode = False

ActiveCell.FormulaR1C1 = "=RC[**-49**]"

Range("**CL1**").Select

Selection.Copy

Range("**CL1:CP1**").Select

ActiveSheet.Paste

ActiveWindow.SmallScroll ToRight:=4

```
    Range("CR1").Select
    Application.CutCopyMode = False
    ActiveCell.FormulaR1C1 = "Resumo"
    Range("CR2").Select
    Application.CutCopyMode = False
    ActiveCell.FormulaR1C1 = "=SUM(RC[-6]:RC[-2])"
    Range("CR2").Select
    Selection.Copy
    Selection.End(xlToLeft).Select
    Selection.End(xlDown).Select
    ActiveCell.Offset(0, 2).Select
    Range(Selection, Selection.End(xlUp)).Select
    ActiveSheet.Paste
    Columns("CR:CR").Select
    Selection.AutoFilter Field:=1, Criteria1:="0"
    Rows("2:2").Select
    Range("CR2").Activate
    Range(Selection, Selection.End(xlDown)).Select
    Selection.Delete Shift:=xlUp
    Range("CR1").Select
    ActiveSheet.ShowAllData
    Selection.AutoFilter
End Sub
```

The first two bold highlights were replaced with the paths to the folders where the files of the federative units corresponding to that specific year were saved. The third bold highlight corresponded to the first cell of the first column after the data. The fourth bold highlight corresponded to the total number of columns with data plus 1.

The code above triggered Macro 2, and all the lines that did not contain at least one of the associated causes were deleted: LINHAA, LINHAB, LINHAC, LINHAD, or LINHAII, starting with “I2.” Then, manually, the process was repeated by applying the formula “INDEX(Sheet1!B:B;MATCH (AO2;Sheet1!B:B;0))”, where “AO2” was the item of LINHAA and “Sheet1!B:B” was the relationship of causes between I20.1 and I25.9. The formula was repeated for the five causes, and all the lines whose five items resulted in error in the formula were then excluded, thus eliminating all the lines that did not have any of the five causes between I20.1 and I25.9.

The bold highlights in Macro 2 indicate items that varied according to each base year. “CL”, corresponds to the third column after the original database, which extends by four additional columns until “CP”. “-49” indicates how many columns must be moved to the left to reach the LINHAA column. Finally, column “CR” is the second column after the five new columns created due to the causes LINHAA to LINHAII. The “CR” column has the function of facilitating the exclusion of all the lines whose five causes do not start in “I2.”

Following that, the asterisks of the subcauses associated with the underlying cause were excluded. Removing the asterisks was critical, as they would confuse the Excel formulas and generate duplicate calculations. Before excluding the asterisks, the initial asterisks (first letter) in the five columns as associated causes were excluded. For that, the following formula was used: IFERROR(RIGHT(CELL;LEN(CELL)-1;””), where CELL was the cell in which the first asterisk had to be cleared. With this, the data was correct and ready for the creation of formulas to start the analysis of recurrences of causes and associated causes.
