# Supplementary material for: Ischemic heart disease-related mortality in Brazil, 2006 to 2020. A study of multiple causes of death
Source: BMC Public Health. 2024 Mar 19;24:849. doi: 10.1186/s12889-024-18162-0 (PMC10949584; doi:10.1186/s12889-024-18162-0)
Supplement: Supplementary file 5 — Supplementary Material 5. [file 12889_2024_18162_MOESM5_ESM.pdf]

**Table S1. Percentage of completion of at least three rows between rows A, B, C, and D and the average number of lines filled per year and geographic region.** The table shows the percentage of at least three lines filled in between lines A, B, C, and D on the death certificate and the average number of lines filled in by year and geographic region.

| | 2006 | 2007 | 2008 | 2009 | 2010 | 2011 | 2012 | 2013 | 2014 | 2015 | 2016 | 2017 | 2018 | 2019 | 2020 |
|---|---|---|---|---|---|---|---|---|---|---|---|---|---|---|---|
| North | 45,58 | 43,38 | 45,07 | 46,01 | 42,32 | 43,56 | 42,08 | 45,94 | 44,62 | 47,76 | 47,1 | 47,13 | 44,21 | 46,84 | 44,33 |
| Northeast | 40,82 | 42,79 | 43,56 | 44,76 | 45,4 | 45,14 | 44,85 | 44,94 | 47,95 | 47,5 | 48,51 | 48,65 | 48,55 | 49,56 | 44,99 |
| Midwest | 44,56 | 47,82 | 48,19 | 49,09 | 46,93 | 48,51 | 48,28 | 51,13 | 52,1 | 54,66 | 54,87 | 53,38 | 53,42 | 54,75 | 50,02 |
| Southeast | 45,51 | 47,27 | 48,18 | 47,79 | 44,46 | 44,76 | 44,57 | 44,73 | 48,57 | 46,87 | 47,28 | 45,37 | 47,14 | 47,76 | 44,64 |
| South | 40,59 | 42,52 | 44,24 | 44,46 | 36,6 | 38,47 | 40,89 | 39,91 | 41,78 | 37,98 | 39,07 | 40,85 | 39,15 | 41,2 | 39,96 |
| | | | | | | | | | | | | | | | |
| North | 2,406 | 2,365 | 2,421 | 2,441 | 2,368 | 2,386 | 2,363 | 2,445 | 2,404 | 2,476 | 2,446 | 2,454 | 2,395 | 2,444 | 2,374 |
| Northeast | 2,307 | 2,356 | 2,373 | 2,404 | 2,414 | 2,421 | 2,414 | 2,405 | 2,467 | 2,446 | 2,476 | 2,481 | 2,466 | 2,497 | 2,227 |
| Midwest | 2,413 | 2,471 | 2,47 | 2,494 | 2,455 | 2,506 | 2,492 | 2,541 | 2,578 | 2,623 | 2,615 | 2,599 | 2,594 | 2,615 | 2,527 |
| Southeast | 2,42 | 2,461 | 2,474 | 2,454 | 2,381 | 2,413 | 2,396 | 2,399 | 2,475 | 2,438 | 2,456 | 2,537 | 2,436 | 2,452 | 2,384 |
| South | 2,3 | 2,346 | 2,387 | 2,379 | 2,182 | 2,249 | 2,303 | 2,283 | 2,319 | 2,233 | 2,282 | 0,409 | 2,277 | 2,314 | 2,285 |
